# National Antibiotic Resistance Monitoring System for Enteric Bacteria

**DOI:** 10.3201/eid1011.040665

**Published:** 2004-11

**Authors:** Cheri N. Holmes, Tom M. Chiller

**Affiliations:** * Centers for Disease Control and Prevention, Atlanta, Georgia, USA

**Keywords:** NARMS, antimicrobial resistance, enteric bacteria, meeting

National Antibiotic Resistance Monitoring System (NARMS)–enteric bacteria is a collaboration by the Centers for Disease Control and Prevention (CDC), the U.S. Food and Drug Administration, the Center for Veterinary Medicine (FDA-CVM), and the U.S. Department of Agriculture, Agricultural Research Services (USDA-ARS). NARMS was established in 1996 and monitors antimicrobial drug resistance in *Campylobacter*, *Escherichia coli* O157:H7, *Enterococcus*, non-Typhi *Salmonella*, *Salmonella* Typhi, and *Shigella*.

The 2004 meeting was held March 4–5 in Decatur, Georgia, and hosted by the Foodborne and Diarrheal Diseases Branch, Division of Bacterial and Mycotic Diseases, National Center for Infectious Diseases, CDC. The meeting highlighted data from scientific studies and surveillance for antimicrobial drug resistance in the United States and abroad with enteric bacteria isolated from humans, animals, and retail foods. Approximately 180 participants from 14 countries, representing 71 organizations, attended the meeting. The organizations included international and national public health agencies, state and local health departments, public health laboratories, industry consumer groups, and academic institutions from Australia, Canada, Cameroon, China, Denmark, Europe, Italy, Japan, Philippines, Poland, Thailand, the United Kingdom, the United States, and Vietnam.

The meeting began with a World Health Organization expert's summarization of a recent workshop on nonhuman antimicrobial drug use and antimicrobial drug resistance. Scientific assessment and risk management of antimicrobial drug use in agriculture and human and veterinary medicine were examined. A plenary session on the human health consequences of antimicrobial drug resistance consisted of two presentations from the United States and two presentations from Denmark.

The results of a study conducted by CDC that found higher rates of hospitalization and death in resistant *Salmonella* infection, as compared to susceptible ones, were presented. The results of another CDC study that found higher frequencies of bloodstream infection and hospitalization with resistant *Salmonella* infections, as compared to susceptible ones, were presented. A presentation from the Statens Serum Institut in Denmark highlighted the association between flouroquinolone-resistant *Campylobacter* infections, as compared to susceptible ones, and a higher frequency of invasive illness, hospitalization, and death. A second presentation demonstrated increased death rates in resistant *S*. Typhimurium infections, as compared to susceptible ones. Other speakers highlighted emerging resistance to clinically important antimicrobial drugs, environmental studies on antimicrobial drug resistance, antimicrobial drug resistance in commensal bacteria, partner perspectives on antimicrobial drug resistance, international perspectives on antimicrobial drug resistance, and NARMS educational activities. A presentation of the "GET SMART: Know When Antibiotics Work on the Farm" campaign highlighted educational efforts to promote the appropriate use of antibiotics in veterinary medicine. The campaign currently includes an interactive Web-based program on aspects of microbiology, pharmacology, infectious disease, and public health for veterinary students and veterinarians who participate in continuing education programs. The conference also included brief summaries of three recent outbreaks of multidrug- resistant *S*. Typhimurium DT104 R-type, which has become a common strain of *Salmonella* isolated from humans and was resistant to ampicillin, chloramphenicol, streptomycin, sulphonamides, and tetracyclines. More information about NARMS and antimicrobial resistance can be found at www.cdc.gov/NARMS.

